# Draft Genome Sequence of the Protozoan Parasite Leishmania braziliensis Strain BA788, Isolated from a Clinical Case in Bahia State, Brazil

**DOI:** 10.1128/mra.00245-22

**Published:** 2022-11-01

**Authors:** Anderson Coqueiro-dos-Santos, Gabrielle Ariadine Bento, Aldina Barral, Camila I. de Oliveira, Daniella C. Bartholomeu

**Affiliations:** a Universidade Federal de Minas Gerais, Instituto de Ciências Biológicas, Laboratório de Imunologia e Genômica de Parasitos, Belo Horizonte, Minas Gerais, Brazil; b Instituto Gonçalo Moniz, Fiocruz, Salvador, Bahia, Brazil; c Instituto Nacional de Ciência e Tecnologia em Doenças Tropicais, Salvador, Bahia, Brazil; Loyola University Chicago

## Abstract

The draft genome of the parasite Leishmania braziliensis strain BA788, which was isolated from a patient from Bahia state, Brazil, was sequenced using Illumina paired-end technology. The assembled genome is 33.5 Mb long and contains 7,603 genes. This genome will contribute to studies aimed at understanding the pathogenesis caused by this parasite strain.

## ANNOUNCEMENT

Leishmaniasis is a complex of diseases transmitted to humans and other mammals by the bite of sand flies from the *Phlebotomus* and *Lutzomyia* genera. These diseases are endemic in 90 countries and pose a risk to 350 million people, with an estimated 1 million cases per year (1). Leishmaniasis encompasses a broad range of clinical manifestations, from localized cutaneous disease to the visceral form with potentially fatal outcomes. Distinct clinical features depend on the *Leishmania* species involved and the host immune response. Leishmania (Viannia) braziliensis is the most important etiological agent of tegumentary leishmaniasis in the Americas, with predominantly rural and peridomiciliary transmission (2).

L. braziliensis strain MHOM/BR/01/BA788 was isolated from a 16-year-old male patient from Jequié, Bahia state, northeastern Brazil, who presented with a single ulcerated lesion on the lower limb (3). The Montenegro skin test supported the diagnosis as localized cutaneous leishmaniasis. Lymph nodes close to the lesion site were aspirated, and samples were cultured in Schneider medium (Sigma Chemical Co.) supplemented with 100 U/mL of penicillin, 100 μg/mL of streptomycin, 10% heat-inactivated fetal calf serum (Life Technologies), and 2% sterile human urine. The research was carried out in accordance with the Declaration of Helsinki. The patient underwent conventional treatment with antimonials, and the lesion was completely reepithelized after a single round of treatment. MHOM/BR/01/BA788 was registered at SisGen (https://sisgen.gov.br/paginas/pubpesqatividade.aspx) under the accession number A7A6463.

Promastigote parasites were maintained in Schneider medium (Sigma Chemical Co.) supplemented with 100 U/mL of penicillin, 100 μg/mL of streptomycin, and 10% heat-inactivated fetal calf serum (Life Technologies). Promastigotes were grown in airtight flasks (T25 [nonvented]), incubated upright in a 25°C incubator, and passaged every 3 days (4). Genomic DNA was extracted using the Wizard genomic DNA purification kit (Promega) following the manufacturer’s instructions. The DNA sample was then checked for *Mycoplasma* contamination by PCR amplification (5) and genotyped by PCR using L. braziliensis-specific primers (6), and its quality and integrity were assessed with ethidium bromide-stained agarose gels. A sequencing library was constructed using the Illumina TruSeq library preparation method. Paired-end sequencing (151 bp from each end) was performed on an Illumina HiSeq 2500 instrument at Macrogen, Inc. (South Korea).

A total of 28,378,882 reads were generated, corresponding to 100× *g*enome coverage. Read quality was assessed using FastQC (http://www.bioinformatics.babraham.ac.uk/projects/fastqc), and low-quality reads and adapter sequences were removed with Trimmomatic v0.39 (7), based on minimal mean Phred quality scores of 30 and read lengths of >50 bp. The reads were mapped against the reference genome of L. braziliensis strain M2904 (GenBank accession number GCA_900537975.1) using BWA-MEM v0.7.17 (8); this resulted in 97.3% mapped reads, which were used for reference-guided assembly with RGAAT v2.0 (9) with the following parameters: –q 20 –l 50 –d 8 –c 5 –fm no. The assembled genome consisted of 33.5 Mb spread over 51 scaffolds, with an *N*_50_ value of 1,142,394 bp and a GC content of 58.05%. The annotation was transferred from L. braziliensis strain M2904 (GenBank accession number GCA_900537975.1) by RGAAT using BLAT v35 genome comparisons (9). The genome contained 7,488 predicted protein-coding genes and 115 structural RNAs. Analyses of the predicted protein sequences with BUSCO v5.2.2 (10) showed that the proteome contained 98.5% of the 130 markers of single-copy ortholog genes that are expected to be present in 31 protozoan species. The annotation was converted to an SQN file by using the NCBI-provided script tbl2asn and was submitted to GenBank.

### Data availability.

This whole-genome shotgun project has been deposited in DDBJ/ENA/GenBank under the accession number JAMFLV000000000. The Illumina raw reads have been deposited in the Sequence Read Archive (SRA) under accession number SRR15902693.
